# Intracranial *Candida* parapsilosis infection occurring 2 years after burr-hole drainage for chronic subdural hematoma: a case report

**DOI:** 10.3389/bjbs.2026.16761

**Published:** 2026-07-29

**Authors:** Junfeng Li, Shaohua Lin, Wei Liu, Pengpeng Li, Lei Luo

**Affiliations:** Department of Neurosurgery, Xi’an Aerospace Hospital of Northwest University, Xi’an, Shaanxi, China

**Keywords:** burr-hole drainage, *Candida* parapsilosis, chronic subdural hematoma, fungal infection, postoperative infection

## Abstract

**Background:**

Chronic subdural hematoma (CSDH) is a common condition affecting patients undergoing neurosurgical treatment for which burr-hole drainage remains the standard treatment. Postoperative fungal infections following this procedure are extremely rare, but can lead to severe consequences.

**Case Description:**

A 66-year-old male with a history of hypertension and schizophrenia underwent bilateral burr-hole drainage for bilateral frontotemporoparietal CSDH at another hospital 2 years prior to presentation. In the intervening years following this procedure, he experienced recurrent headaches, developed involuntary tongue protrusion and limb shaking, and suffered one episode of seizure 10 days before admission. Imaging suggested recurrence and possible organization of the bilateral CSDH. Bilateral craniotomy for hematoma evacuation was performed. Intraoperative examination revealed the organization and thickening of the subdural hematoma membrane, with a maximum thickness of approximately 1 cm. Postoperative pathological examination unexpectedly revealed structures suggestive of fungal hyphae. Subsequent cerebrospinal fluid culture confirmed the presence of *Candida* parapsilosis infection, establishing the definitive etiological diagnosis. The patient was successfully cured through surgical evacuation, antifungal therapy consisting of sequential voriconazole and fluconazole treatment, temporary lumbar drainage, and comprehensive management of complications, although the course of hospitalization was further complicated by intestinal obstruction, pneumonia, and bacteremia.

**Conclusion:**

This report describes a rare case of intracranial C. parapsilosis infection occurring 2 years after burr-hole drainage for the management of a CSDH. This case highlights the diagnostic challenges associated with delayed postoperative fungal infections and emphasizes the importance of pathological examination when encountering atypical surgical findings. A multidisciplinary approach combining aggressive surgical debridement with targeted antifungal therapy is crucial for achieving a favorable outcome.

## Introduction

Chronic subdural hematoma is a common condition affecting patients undergoing neurosurgery and is particularly prevalent among older adults. Burr-hole drainage has become the preferred treatment for CSDH, as it is a relatively simple, minimally invasive procedure with a generally good safety profile. Secondary intracranial fungal infection following CSDH surgery is exceedingly rare. Fungal infections of the central nervous system (CNS) are among the diseases with the highest global morbidity and mortality, but are relatively uncommon, accounting for less than 5% of all CNS infections [1]. In several retrospective cohorts, Aspergillus species were identified as a leading cause of fungal CNS infections [2, 3], and they are the primary pathogens associated with infections following neurosurgical procedures—particularly transsphenoidal surgery—potentially from Aspergillus contamination of the paranasal sinuses [4]. Morioka et al. previously reported a case of cerebral aspergillosis occurring 2.5 years after burr-hole drainage for CSDH, with a clinical course resembling a brain tumor [5]. *Candida* parapsilosis is an important non-albicans *Candida* species that has been increasingly reported as a cause of infections among immunocompromised patients, neonates, and patients with indwelling devices in recent years [6]. This species has a particular affinity for artificial materials and can form biofilms on plastic surfaces, making it a significant cause of device-associated infections [7]. C. parapsilosis has been found to cause CNS infections, including meningitis, ventriculitis, and brain abscesses [8, 9]. Device-related CNS infections caused by C. parapsilosis have been reported in both infants and adults, with amphotericin B or fluconazole comprising standard treatment regimens for affected patients [10]. To the best of our knowledge, no previous report has described intracranial C. parapsilosis infection occurring years after burr-hole surgery for CSDH. Here, we present such a case, diagnosed 2 years postoperatively. Diagnosis was established through pathological examination of the organized hematoma membrane obtained during the second surgery and subsequent CSF culture. This case report and associated literature review aim to detail the clinical characteristics, diagnostic pitfalls, and treatment strategies for this rare but serious complication.

## Case description

The patient was a 66-year-old male with a history of hypertension and schizophrenia. He had no history of immunodeficiency disorders and had not received glucocorticoids or immunosuppressive agents. Two years prior to presentation, he underwent bilateral burr-hole drainage at another hospital for management of bilateral frontotemporoparietal CSDH. Intraoperative drainage was limited, and his postoperative course was uneventful, with no fever incidence. The drain was removed 48 h post-surgery, and good wound healing was observed, with eventual discharge after symptomatic improvement. Over the 2 years following this procedure, the patient experienced recurrent, progressively worsening headaches, accompanied by involuntary tongue protrusion and limb shaking. His body temperature remained normal. Ten days before admission to our hospital, he suffered a generalized tonic-clonic seizure that resolved spontaneously after approximately two minutes.

**FIGURE 1:**
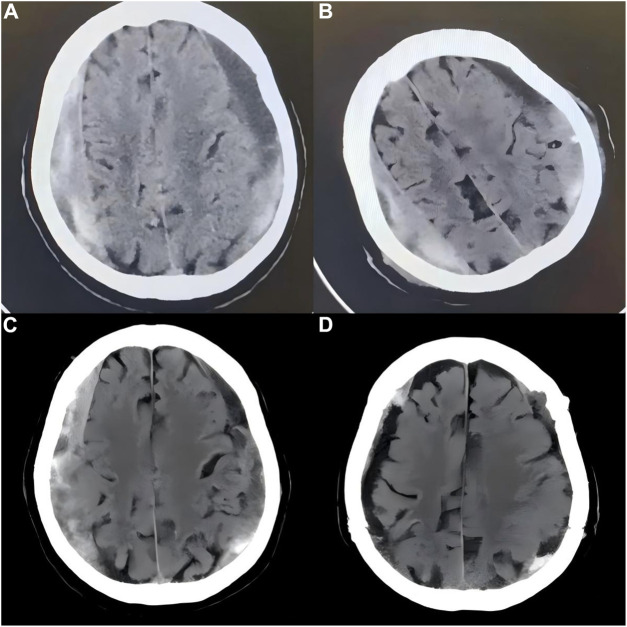
**(A)** CT scan performed before the initial burr-hole drainage surgery 2 years prior, showing hyperdense areas. **(B)** CT scan performed 1 week after the initial burr-hole drainage procedure, showing persistent bilateral subdural collections. **(C)** Preoperative cranial CT scan before craniotomy, showing bilateral frontotemporoparietal chronic subdural hematomas of inhomogeneous density, including hyperdense areas and a significant mass effect. **(D)** Follow-up CT scan on postoperative day 40, showing good re-expansion of the brain parenchyma in the operative area.

**FIGURE 2:**
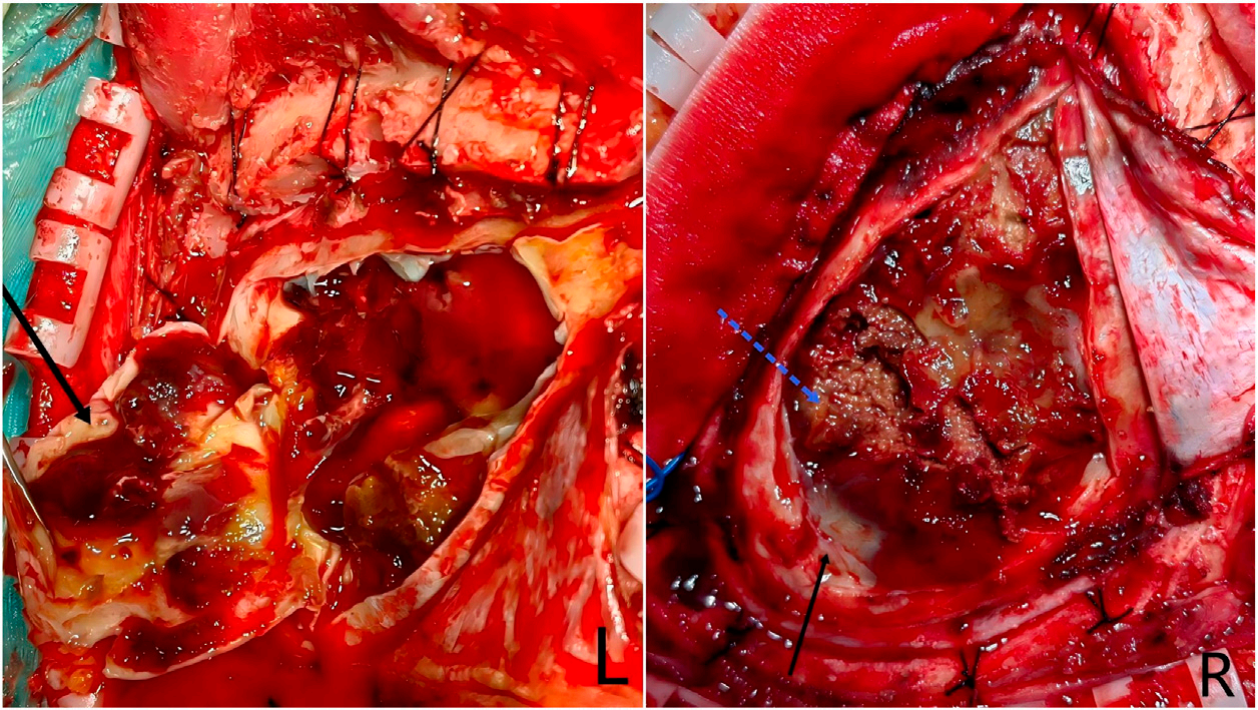
Intraoperative view during the craniotomy procedure. After opening the dura mater and incising the outer membrane of the hematoma, contents with a sandy-like consistency were visible (blue dashed arrow), with significant localized thickening of the hematoma membrane (black arrow).

Review of computed tomography (CT) scans performed at the other hospital before and after the initial surgery 2 years earlier revealed persistent bilateral hematomas (Figures 1A,B). Upon admission, cranial CT revealed bilateral frontotemporoparietal CSDH of inhomogeneous density, including hyperdense areas, and a significant mass effect (Figure 1C). These hyperdense areas were suspected to represent the organization of the chronic hematoma. Given the patient’s progressive symptoms, which were attributed to persistent brain compression caused by the organized hematoma, it was concluded after discussion that burr-hole drainage or conservative management would not effectively drain the hematoma or relieve brain compression. Preoperative evaluation revealed no surgical contraindications, with serum immunoglobulin levels (IgG, IgA, IgM) that were within normal ranges. After appropriate preoperative preparation, the patient underwent bilateral frontotemporoparietal craniotomy for hematoma evacuation under general anesthesia. Intraoperatively, the hematoma membrane was found to be abnormally thickened and organized, measuring up to 1 cm at its thickest point, with a leathery, tough consistency. The cavity contained a small amount of yellowish fluid and abundant organized material with a “sandy” appearance (Figure 2). These intraoperative findings differed from a typical CSDH case such that the hematoma membrane and its contents were sent for postoperative pathological examination. Craniotomy achieved substantial removal of the membrane and cavity contents.

**FIGURE 3:**
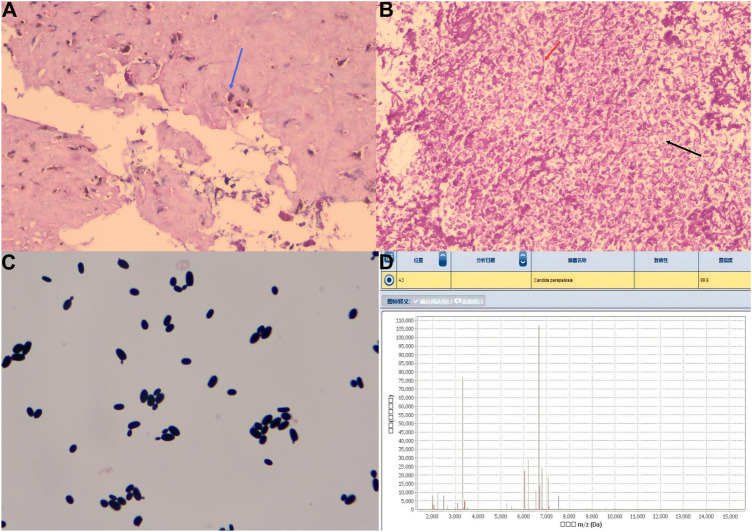
**(A)** Postoperative pathological examination revealed fibrous connective tissue with hyaline degeneration and hemosiderin deposition (blue arrow). **(B)** Fungal hyphae (red arrow) and fungal spores (black arrow) were visible on pathological examination. **(C)** CSF culture revealed fungal spores visible under microscopy. **(D)** Matrix-assisted laser desorption ionization time-of-flight mass spectrometry (MALDI-TOF MS) results were positive for *Candida* parapsilosis.

Postoperatively, the patient was transferred to the intensive care unit (ICU) with a low-grade fever and a decreased level of consciousness (lethargy). Empirical antibiotic therapy with ceftriaxone was initiated. The intraoperatively placed subdural drain drained approximately 200 mL of hemorrhagic fluid daily and was removed on postoperative day (POD) 3. On POD 5, histopathological examination of the resected membrane revealed fibrous connective tissue with hyaline degeneration, hemosiderin deposition, and structures suggestive of fungal hyphae on hematoxylin and eosin (H&E) staining (Figures 3A,B); however, special fungal stains, including Grocott’s methenamine silver (GMS) and periodic acid-Schiff (PAS) staining, were not performed due to technical constraints at the treating institution. Given these atypical findings, a multidisciplinary team meeting was convened. Then a lumbar puncture was performed, and CSF analysis revealed a total nucleated cell count of 1,098 × 10^6^/L, a white blood cell (WBC) count of 98 × 10^6^/L, 9.2% mononuclear cells, and 90.8% polymorphonuclear cells. Serum (1→3)-β-D-glucan testing was positive. Empirical antifungal therapy with voriconazole was initiated. In light of the potential for fungal dissemination within the now widely opened subdural cavity, a lumbar drain was placed for continuous CSF drainage.

On POD 6, follow-up magnetic resonance imaging (MRI) revealed reduction of the subdural hematoma, with no definitive evidence of intraparenchymal abscess or meningitis. To exclude hematogenous fungal dissemination, three serial blood culture sets were performed at 24-h intervals. Each set was inoculated concurrently into aerobic, anaerobic, and fungal blood culture bottles. After an extended incubation period of 14 days, all cultures were negative for fungal growth, effectively ruling out concomitant fungemia. To exclude the possibility of contamination during the sampling process, sterile saline was used as a negative control in all fungal cultures. Furthermore, only those isolates that were recovered from at least two independent specimens were considered to represent true pathogens. Microbiological identification of the fungal pathogen was performed by initially inoculating the CSF specimen onto Sabouraud dextrose agar and incubating the samples at 35 °C. After 48 h, colonies were observed with a creamy-white, smooth, glistening appearance. Gram staining revealed ovoid budding yeast-like cells. Further identification was performed using matrix-assisted laser desorption ionization-time of flight mass spectrometry (MALDI-TOF MS) (bioMérieux, Marcy-l'Étoile, France). The isolate was identified as *Candida* parapsilosis at a confidence level of 99.9%. Direct microscopic examination of the CSF sediment after Gram staining also revealed the presence of fungal spores. Due to the low volume of the CSF specimen, antifungal susceptibility testing was not performed. On POD 8, CSF culture revealed fungal spores visible under microscopy, and MALDI-TOF MS results were positive for C. parapsilosis (Figures 3C,D). Antifungal therapy was then switched to targeted treatment with fluconazole (400 mg daily). The patient developed diarrhea, which was attributed to enteral nutrition rather than fluconazole. To prevent secondary intracranial infection, the lumbar drain was removed after 5 days. A repeat lumbar puncture on POD 12 showed significant improvement in CSF parameters, with an intracranial pressure (ICP) of 120 mmH_2_O, a WBC count of 8 × 10^6^/L (75% mononuclear cells), glucose levels at 1.99 mmol/L, and protein levels at 3,086 mg/L. Subsequent CSF cultures were negative.

**FIGURE 4:**
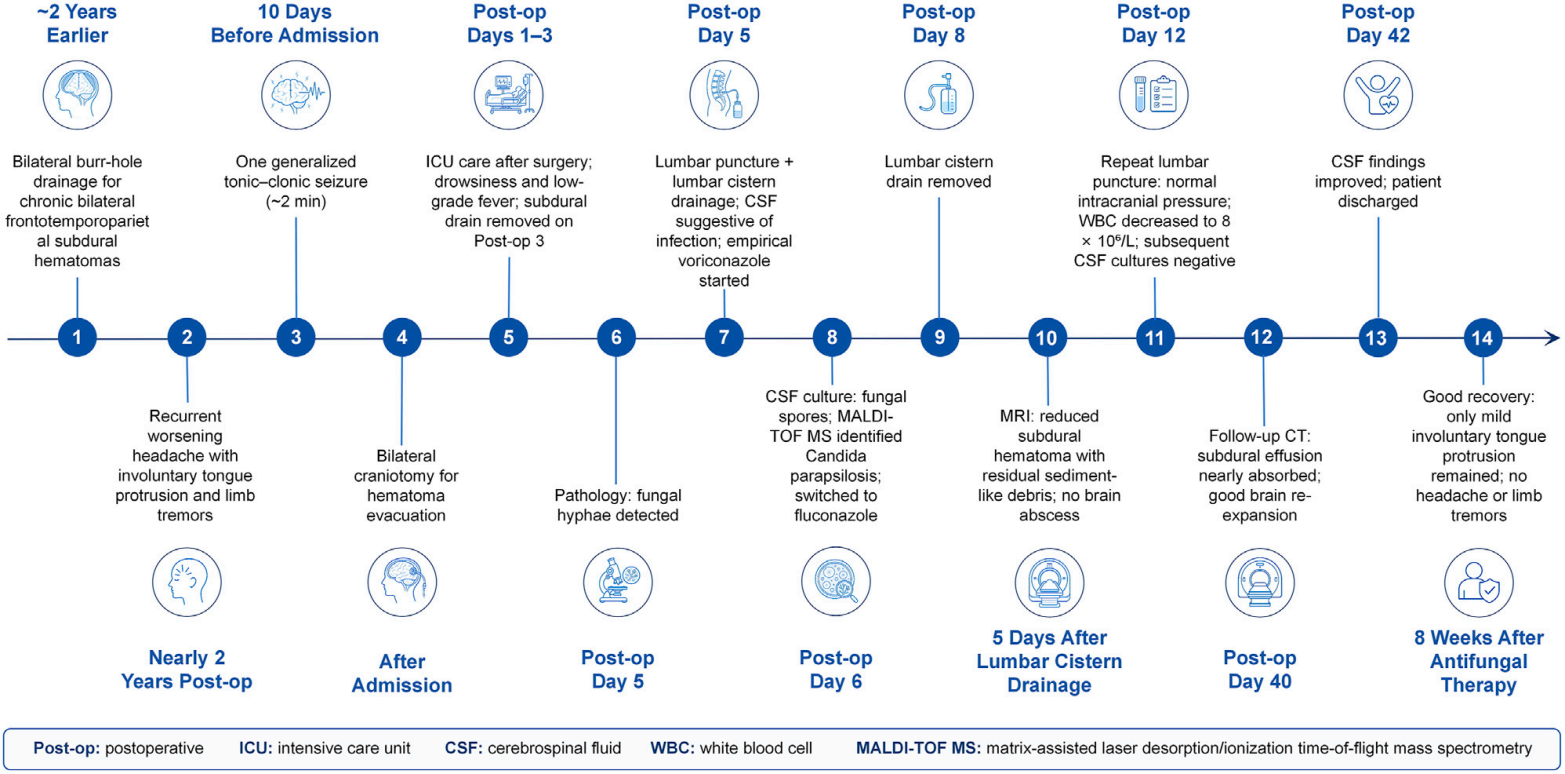
Timeline of the treatment course.

The patient’s hospital course was complicated by abdominal distension, paralytic ileus, pneumonia, and bacteremia, all of which were successfully managed with supportive care and targeted antibiotics. A follow-up CT scan on POD 40 showed near-complete absorption of the subdural fluid collection and good re-expansion of the brain parenchyma in the operative area (Figure 1D). As of POD 42, CSF parameters had continued to improve. After completing 8 weeks of antifungal therapy, the patient was discharged with only mild residual involuntary tongue protrusion and no headaches or limb shaking. At 3 months post-discharge, the patient remained asymptomatic, with no recurrence of headache or seizure, and no clinical or radiological evidence of recurrent infection. A summary of the patient’s clinical course is presented in Figure 4.

## Discussion

This case report presents a rare instance of intracranial C. parapsilosis infection discovered 2 years after burr-hole drainage for CSDH management, with a diagnostically challenging course. The rarity and delayed presentation of this case prompted us to systematically review the literature pertaining to postoperative fungal infections of the CNS. Previous studies have identified *Candida* albicans and C. parapsilosis as the predominant pathogens associated with postoperative *Candida* CNS infections, with common risk factors including prolonged indwelling drainage, broad-spectrum antibiotic use, and immunocompromised status [11]. A 12-year retrospective review of post-neurosurgical *Candida* CNS infections revealed that, despite their extremely low incidence, the associated mortality rate exceeds 30%, and diagnostic delays are common [12]. The pathogenic mechanisms underlying iatrogenic fungal CNS infections have been systematically delineated, highlighting surgery, indwelling devices, and contaminated materials as the three principal sources of pathogen introduction [13]. Among published reports, the case most analogous to ours involved an adult patient with a C. parapsilosis ventriculoperitoneal (VP) shunt infection, which similarly necessitated device removal and extended antifungal therapy [14]. Another autopsy-confirmed case of C. albicans meningoencephalitis following vestibular schwannoma surgery presented with an indolent course and ultimately proved fatal owing to delayed diagnosis, underscoring the critical importance of early pathological diagnosis, as achieved in our patient [15]. Additionally, a case of cerebral aspergillosis occurring 2.5 years after burr-hole drainage for CSDH has been described, which closely parallels our case in terms of latency and surgical approach, albeit with a different pathogen [5]. Other reports have documented instances of brain abscess and shunt-related meningitis caused by C. parapsilosis [8, 16]. To our knowledge, this is the first reported case of intracranial C. parapsilosis infection diagnosed 2 years after burr-hole drainage for CSDH, with confirmation by both histopathology and CSF culture.

The clinical presentation in this case was insidious and non-specific, characterized by progressive headache, movement disorder, and a single seizure, without overt signs of infection such as high fever. This indolent course is typical of chronic fungal infections and can easily be mistaken for recurrence or organization of the original hematoma [5]. The 2-year latency period is particularly noteworthy, as it suggests that fungal elements can remain dormant within the hematoma cavity, which may offer an immune-privileged microenvironment that provides protection against host immune-mediated detection. The key to diagnosis in this case was histopathological examination of the surgically excised membrane. Although preoperative imaging suggested organization, it was unable to distinguish between a sterile organized hematoma and an infected one. Atypical findings—such as an abnormally thickened or granulomatous membrane—should prompt immediate submission of tissue for pathological and microbiological studies, including fungal staining and culture [5, 9]. CSF analysis and culture provided postoperative diagnostic confirmation. Initial CSF profiling revealed marked polymorphonuclear pleocytosis, elevated protein levels, and low glucose, consistent with infection but not specific for fungal infection. When feasible, CSF testing for (1→3)-β-D-glucan can provide rapid screening for fungal infection and has significant value for preemptive diagnosis of iatrogenic fungal CNS infections [10]. Definitive identification relied on CSF culture and MALDI-TOF MS confirmation of C. parapsilosis.

Several conditions may present with similar clinical and imaging features, including recurrent organized chronic subdural hematoma (OCSH) without infection, inflammatory membrane formation, subdural empyema, neoplastic processes, and sterile postoperative changes [17]. Recurrent OCSH is a rare subtype of CSDH with an incidence of 0.3%–6.5% among all chronic subdural hematoma cases, typically lacking systemic signs of infection and showing progressive enlargement with internal septations on imaging [18, 19]. Histopathologically, the outer membrane evolves through progressive fibrosis mediated by transforming growth factor-beta (TGF-β) signaling [20]. In this patient, the 2-year latency and absence of fever were initially suggestive of simple organization, but the leathery membrane with sandy material was not pathognomonic. Definitive diagnosis relied on histopathology revealing fungal hyphae and CSF culture positive for C. parapsilosis [5]. Inflammatory membrane formation secondary to hemorrhage or prior surgery may cause fibrotic thickening [20], but could not explain the fungal elements [21]. Subdural empyema is a rare neurosurgical emergency with mortality as high as 28% [22, 23], most often presenting acutely with fever and rapid deterioration. In contrast, our patient’s indolent 2-year course, absence of purulent exudate, and CSF culture positive for C. parapsilosis but negative for bacterial growth excluded this diagnosis [24]. Neoplastic processes were ruled out by follow-up MRI and histopathological examination [25, 26]. Sterile postoperative changes usually occur within weeks to months; however, delayed fungal infection has been reported [27]. Progressive worsening over 2 years with positive culture confirmed active infection.

C. parapsilosis is a common commensal of the human skin with a well-characterized ability to form biofilms on medical devices, making it an important pathogen in healthcare-associated infections [6]. CNS infections caused by this organism are very rare, with previous reports primarily focusing on shunt-related or post-neurosurgical infections [7, 9, 10]. Bhalla et al. described device-associated CNS C. parapsilosis infection in a 6-month-old infant with hydrocephalus [9]. Basson et al. reported the first case of C. parapsilosis brain abscess in a 15-year-old female [8]. The present case expands the known clinical spectrum, demonstrating that C. parapsilosis can cause chronic infection localized within an organized subdural hematoma years after surgery, even without long-term implant retention. Based on the clinical features and supporting literature, we propose a multifactorial model for the 2-year latency. The most likely source is iatrogenic contamination during initial burr-hole drainage, as neurosurgical procedures are the most common iatrogenic route for fungal entry into the CNS [13]. C. parapsilosis may gain access via the patient’s skin, surgeon’s gloves, or contaminated instruments [6]. The hematoma cavity is relatively enclosed and lacks effective immune surveillance, allowing even a small inoculum to establish a protected niche [10]. Although drain placement lasted only 48 h, C. parapsilosis can form biofilms on plastic surfaces [9, 14]. Biofilm fragments or colonized fungi can persist after drain removal and serve as a reservoir for late-onset infection [12]. The chronic subdural hematoma cavity constitutes a hypoxic, iron-rich microenvironment that suppresses local immunity, and the thickened membrane (up to 1 cm) physically sequesters fungi, protecting them from host immune surveillance and antifungal agents. This explains the patient’s 2-year asymptomatic course, with no fever and negative blood cultures, and underscores why diagnosis was only established after membrane resection—mirroring previous reports of occult fungal proliferation [15]. Although direct evidence is lacking, preventable iatrogenic factors—such as inadequate skin antisepsis or contamination during drain care—cannot be excluded. Strict adherence to aseptic protocols, minimization of indwelling device duration, and avoidance of contamination are core preventive measures [13].

Managing intracranial fungal infections requires combined medical and surgical approaches. Craniotomy served dual purposes: mass effect relief and reduction of fungal burden by resecting the infected membrane. Antifungal selection should be guided by species identification and susceptibility testing, when possible. Voriconazole offers broad-spectrum coverage, but for fluconazole-susceptible C. parapsilosis, fluconazole is the drug of choice, offering good CNS penetration [6, 9]. Amphotericin B has limited CNS access [8]. Lumbar drainage reduces fungal load and enables response monitoring, though it carries an infection risk [9]. Antifungal therapy alone is often insufficient; surgical resection of infected membranes and removal of contaminated devices are essential [11, 12]. Similar success has been reported with device removal and fluconazole for shunt-related C. parapsilosis infections [14]. A fatal case was attributed to diagnostic delay [15]. Despite systemic complications, a favorable outcome in the present case resulted from multidisciplinary collaboration [10]. Risk factors include immunocompromised status, diabetes, corticosteroids, malignancy, recent neurosurgery, and indwelling devices [6, 9, 10]. Prevention focuses on strict aseptic technique, minimizing drain duration, and avoiding contamination [10]. Our patient had normal immunoglobulin levels and no immunosuppressive therapy, suggesting surgery itself was the primary risk factor. This single case report has inherent limitations. These findings cannot be generalized. The exact route of infection could not be determined. Antifungal susceptibility testing was not performed. Therapy was based on empirical considerations, supported by: (1) C. parapsilosis isolates generally being fluconazole-susceptible [6, 28]; (2) fluconazole achieving CSF concentrations approximately 70%–80% of serum levels [29]; and (3) favorable clinical and microbiological response. Lack of susceptibility testing further limits generalizability. GMS and PAS staining were not performed due to technical constraints. Diagnosis was supported by H&E findings suggestive of fungal hyphae and confirmed by CSF culture and MALDI-TOF MS. Biofilm formation assays and molecular typing were not performed. Long-term follow-up is required to monitor for late recurrence.

In summary, we report the first case of intracranial *Candida* parapsilosis infection occurring 2 years after burr-hole drainage for CSDH. This case highlights the diagnostic challenges of delayed postoperative fungal infections, which can masquerade as hematoma organization, and underscores the critical role of histopathological examination when atypical specimens are encountered. A multidisciplinary approach combining surgical debridement with prolonged antifungal therapy is key to a favorable outcome. This case represents a biomedical advance as the first evidence that C. parapsilosis can establish a latent intracranial infection within an organized subdural hematoma cavity for up to 2 years post-surgery—extending the recognized latency period for non-albicans *Candida* CNS infections and establishing histopathological examination of atypical surgical specimens as the essential diagnostic clue that should prompt directed microbiological testing in such delayed presentations.

## Data Availability

The original contributions presented in the study are included in the article/supplementary material, further inquiries can be directed to the corresponding author.

## References

[1] Bora S Kumar A Mishra S Satyarthee GD Singh PK Sawarkar D Intracranial aspergillosis amongst immunocompetent patients: an experience with combined surgical and medical management of 18 patients. Clin Neurol Neurosurg (2019) 186:105511. 10.1016/j.clineuro.2019.105511 31505434

[2] Grant LM Vega PJT Yaman RN Girardo ME Beam E Razonable RR Brain abscess following solid organ transplantation: a 21-year retrospective study. Transpl Infect Dis (2024) 26(6):e14394. 10.1111/tid.14394 39400917

[3] Bogaart L Lang BM Rossi S Neofytos D Walti LN Khanna N Central nervous system infections in solid organ transplant recipients: results from the Swiss transplant cohort study. J Infect (2022) 85(1):1–7. 10.1016/j.jinf.2022.05.019 35605804

[4] Pasqualotto AC Denning DW . Post-operative aspergillosis. Clin Microbiol Infect (2006) 12(11):1060–76. 10.1111/j.1469-0691.2006.01512.x 17002605

[5] Morioka T Tashima T Nagata S Fukui M Hasuo K . Cerebral aspergillosis after burr-hole surgery for chronic subdural hematoma: case report. Neurosurgery (1990) 26(2):332–5. 10.1097/00006123-199002000-00026 2308684

[6] Trofa D Gácser A Nosanchuk JD . Candida parapsilosis, an emerging fungal pathogen. Clin Microbiol Rev (2008) 21(4):606–25. 10.1128/CMR.00013-08 18854483 PMC2570155

[7] Pfaller MA Diekema DJ . Epidemiology of invasive candidiasis: a persistent public health problem. Clin Microbiol Rev (2007) 20(1):133–63. 10.1128/CMR.00029-06 17223626 PMC1797637

[8] Basson D Naidoo TK Elliott E Saul-Macala Y He WD Thomas A . Candida parapsilosis intracerebral abscess and intralesional amphotericin B: a novel treatment approach to a rare infection. Illustrative case. J Neurosurg Case Lessons (2024) 8(5):CASE2484. 10.3171/CASE2484 39074395 PMC11301601

[9] Bhalla GS Malik M Sarao MS Bandyopadhyay K Singh P Tadepalli S Device-associated central nervous system infection caused by Candida parapsilosis. Cureus (2018) 10(8):e3140. 10.7759/cureus.3140 30345197 PMC6188172

[10] Chiu C-Y Hicklen RS Ostrosky-Zeichner L Sheth SA Tan C Weinberg JS An unintended consequence: a review of iatrogenic central nervous system mold infections and outbreaks. Clin Microbiol Rev (2026) 39(1):e0028225. 10.1128/cmr.00282-25 41665346 PMC12981134

[11] Chen H Cong W Xie D Wang S Niu J Chen G Candida central nervous system infection after neurosurgery: a single-institution case series and literature review. Ann Palliat Med (2021) 10(11):11362–9. 10.21037/apm-21-2577 34872262

[12] O'Brien D Stevens NT Lim CH O'Brien DF Smyth E Fitzpatrick F Candida infection of the central nervous system following neurosurgery: a 12-year review. Acta Neurochir (Wien) (2011) 153(6):1347–50. 10.1007/s00701-011-0990-9 21431456

[13] Lahoti S Joseph RB . Iatrogenic fungal infections of central nervous system. Curr Neurol Neurosci Rep (2013) 13(11):399. 10.1007/s11910-013-0399-3 24078440

[14] Fadel H Moon S-J Klinger NV Chamiraju P Eltahawy HA Moisi MD Candida parapsilosis infection of ventriculoperitoneal shunt in adult: case report and literature review. World Neurosurg (2018) 119:290–3. 10.1016/j.wneu.2018.08.023 30114539

[15] Camatti J Tudini M Bonasoni MP Santunione AL Cecchi R Radheshi E Candida albicans meningoencephalitis after vestibular schwannoma surgery: an autopsy-confirmed case report. Diagnostics (Basel) (2026) 16(2):228. 10.3390/diagnostics16020228 41594204 PMC12839699

[16] Bagheri F Kelly LC Maruf M Marino W Santucci T Jr . Candida parapsilosis meningitis associated with shunt infection in an adult Male. Clin Neurol Neurosurg (2010) 112(3):248–51. 10.1016/j.clineuro.2009.11.011 20022423

[17] Catana D Koziarz A Cenic A Nath S Singh S Saleh AA Subdural hematoma mimickers: a systematic review. World Neurosurg (2016) 93:73–80. 10.1016/j.wneu.2016.05.084 27268313

[18] Cao J Wang Z Liu C Shen J Fang J . Organized chronic subdural hematoma mimicking acute epidural hematoma. Case Rep Med (2023) 2023:6645752. 10.1155/2023/6645752 38053805 PMC10695690

[19] Lee K-S . History of chronic subdural hematoma. Korean J Neurotrauma (2015) 11(2):27–34. 10.13004/kjnt.2015.11.2.27 27169062 PMC4847516

[20] Ryu HS Sun Kim S Hong WJ Kim T-S Joo S-P . Differences in gross appearance and histopathology of the outer membrane of the subdural hematoma envelope over time: a respective case series and literature review. Medicine (Baltimore) (2023) 102(29):e34257. 10.1097/md.0000000000034257 37478245 PMC10662858

[21] Bokka S Trivedi A . Histopathological study of the outer membrane of the Dura mater in chronic sub dural hematoma: its clinical and radiological correlation. Asian J Neurosurg (2016) 11(1):34–8. 10.4103/1793-5482.154979 26889276 PMC4732239

[22] Madan A Wilson M Garcia A Hamam F Rhandhawa R. Subdural empyema as a sequela of severe erosive sinusitis: a case report. Cureus (2024) 16(8):e67690. 10.7759/cureus.67690 39314621 PMC11419590

[23] Arbuckle BB Abo Kasem R Shaik A Downes A Hwang S Passias PG Microbial analyses of subdural empyema: a case report and literature review. Cureus (2025) 17(1):e76800. 10.7759/cureus.76800 39897219 PMC11786810

[24] John E . Greenlee. Subdural empyema. Curr Treat Options Neurol (2003) 5(1):13–22. 10.1007/s11940-003-0019-7 12521560

[25] Bordia R Le M Behbahani S . Pitfalls in the diagnosis of subdural hemorrhage - mimics and uncommon causes. J Clin Neurosci (2021) 89:71–84. 10.1016/j.jocn.2021.02.006 34119298

[26] Hamamoto R Kawasaki T Oda M Sumiyoshi S Hayashi K Kobayashi T Primary extranodal marginal zone mucosa-associated lymphoid tissue-type B-cell lymphoma involving the dura: a case report. Surg Neurol Int (2024) 15:113. 10.25259/SNI_792_2023 38628522 PMC11021089

[27] O'Brien D Cotter M Lim CH Sattar MT Smyth E Fitzpatrick F . Candida parapsilosis meningitis associated with gliadel (BCNU) wafer implants. Br J Neurosurg (2011) 25(2):289–91. 10.3109/02688697.2010.534202 21158511

[28] Pfaller MA Diekema DJ Turnidge JD Castanheira M Jones RN . Twenty years of the SENTRY antifungal surveillance program: results for candida species from 1997–2016. Open Forum Infect Dis (2019) 6(Suppl. 1):S79–S94. 10.1093/ofid/ofy358 30895218 PMC6419901

[29] Felton T Troke PF Hope WW . Tissue penetration of antifungal agents. Clin Microbiol Rev (2014) 27(1):68–88. 10.1128/CMR.00046-13 24396137 PMC3910906

